# Synthesis and Evaluation of a Chitosan Oligosaccharide-Streptomycin Conjugate against *Pseudomonas aeruginosa* Biofilms

**DOI:** 10.3390/md17010043

**Published:** 2019-01-10

**Authors:** Ruilian Li, Xianghua Yuan, Jinhua Wei, Xiafei Zhang, Gong Cheng, Zhuo A. Wang, Yuguang Du

**Affiliations:** 1 University of Chinese Academy of Sciences, Beijing 100049, China; rlli@ipe.ac.cn; 2 Key Laboratory of Biopharmaceutical Production & Formulation Engineering, PLA and State Key Laboratory of Biochemical Engineering, Institute of Process Engineering, Chinese Academy of Sciences, Beijing 100190, China; jhwei@ipe.ac.cn (J.W.); gcheng@ipe.ac.cn (G.C.); 3 College of Life Science, Sichuan Normal University, Chengdu 610101, China; lemonlyty@sohu.com (X.Y.); feifei_2016@126.com (X.Z.)

**Keywords:** chitosan oligosaccharides, streptomycin, *Pseudomonas aeruginosa*, biofilms, conjugation

## Abstract

Microbial biofilms are considerably more resistant to antibiotics than planktonic cells. It has been reported that chitosan coupling with the aminoglycoside antibiotic streptomycin dramatically disrupted biofilms of several Gram-positive bacteria. This finding suggested the application of the covalent conjugate of antimicrobial natural polysaccharides and antibiotics on anti-infection therapy. However, the underlying molecular mechanism of the chitosan-streptomycin conjugate (CS-Strep) remains unclear and the poor water-solubility of the conjugate might restrict its applications for anti-infection therapy. In this study, we conjugated streptomycin with water-soluble chitosan oligosaccharides (COS). Unlike CS-Strep, the COS-streptomycin conjugate (COS-Strep) barely affected biofilms of tested Gram-positive bacteria. However, COS-Strep efficiently eradicated established biofilms of the Gram-negative pathogen *Pseudomonas aeruginosa*. This activity of COS-Strep was influenced by the degree of polymerization of chitosan oligosaccharide. The increased susceptibility of *P. aeruginosa* biofilms to antibiotics after conjugating might be related to the following: Suppression of the activation of MexX-MexY drug efflux pump system induced by streptomycin treatment; and down-regulation of the biosynthesis of biofilm exopolysaccharides. Thus, this work indicated that covalently linking antibiotics to chitosan oligosaccharides was a possible approach for the development of antimicrobial drugs against biofilm-related infections.

## 1. Introduction

Biofilms are a form of existence of microorganisms encapsulated in an extracellular matrix that holds cells together and forms a three-dimensional structure against environmental challenges [1]. Recent studies indicated the antimicrobial resistance in bacteria was closely related to the formation of biofilms, which is associated with about 75% of human bacterial infections [2,3,4,5]. The drug-resistance of bacteria in biofilms raised 10–1000 times compared to their planktonic counterparts [6,7,8]. Several factors have been attributed to the resistance, including hampered penetration of antimicrobials through the biofilm matrix, upregulation of the drug efflux pump system, etc. [9,10]. There are limited drugs specifically targeting the biofilms of bacteria. The development of biofilm-specific drugs to treat biofilm-related infections is urged.

Chitosan coupled with streptomycin (CS-Strep) could efficiently disrupted the pre-formed bacterial biofilms. However, the anti-biofilm specificity of CS-Strep was likely restricted to Gram-positive organisms such as *Staphylococcus aureus*, *Listeria monocytogenes*, *Enterococcus faecalis*, but not Gram-negative bacteria *Pseudomonas aeruginosa* [11,12]. These findings suggested an innovative strategy to combat biofilm-related infections by conjugating antibiotics with natural polysaccharides. However, the mechanism behind this intriguing activity is still unclear. The low water solubility and high viscosity of chitosan and CS-Strep might restrict their applications on anti-biofilm therapies. 

Chitosan oligosaccharides (COS), composed of β-(1-4)-linked D-glucosamine and *N*-acetyl-D-glucosamine with the degree of polymerization (DP) 2–10, were prepared from degradation of chitosan. Compared to chitosan, COS have a much lower average molecular weight (MW) and better water-solubility [13]. COS also possess versatile biological activities, such as antimicrobial, antioxidant, anticancer, and immune-stimulant effects [14]. In this study, we coupled streptomycin with COS. The anti-biofilm activities of the COS-streptomycin conjugate (COS-Strep) against bacterial biofilms were evaluated. The structure and function relationships of the anti-biofilm activities were preliminarily explored. 

## 2. Materials and Methods

### 2.1. Reagents and Bacteria Strains

COS (DP 2~8, average MW: 835) and COS2 (DP 2~20, average MW: 1419) were purchased from GlycoBio (GlycoBio, Dalian, China). The average MW and DP of COS was determined by HPLC and LC-MS [15] (Figure S1). Chitosan with MW of 50–190 kD was purchased from Sigma (Sigma-Aldrich, St. Louis, MO, USA). Streptomycin (Strep) was purchased from Solarbio (Solarbio, Beijing, China). The *P. aeruginosa* (PAO1) strain used in the experiment was generously granted by Prof. Ma Lvyan. The *S. aureus* strain (CGMCC1.2910) was purchased from China General Microbiological Culture Collection Center (CGMCC). *L. monocytogenes* (CVCC1597) was purchased from China Veterinary Culture Collection Center (CVCC). *P. aeruginosa* and *L. monocytogenes* were cultured with LB medium at 28 or 37 °C respectively. *S. aureus* was cultured with TSB medium at 37 °C. 

### 2.2. Synthesis of Chitosan Oligosaccharide–Streptomycin Conjugates

The synthesis of the conjugates was based on the oxidation-reduction reaction between COS and streptomycin. The COS-Strep conjugates were prepared as previously described [16]. Briefly, 100 mg COS was first dissolved in 2 mL deionized water, then the pH was adjusted to 4.0 with 0.2 M acetic acid. Then, 525 mg streptomycin was mixed with COS solution at 35 °C with stirring for 1 h in the dark. The synthesis reaction was initialized by the addition of 1 mL 113 mg/mL NaCNBH_3_ with stirring for 24 h and terminated with 2 M NaOH. The solution was then dialyzed (molecular weight cut off: 500–1000 Da) with deionized water for 48 h. A total of 120 mg of COS-Strep conjugates was collected after lyophilization. 

### 2.3. Matrix-Assisted Laser Desorption/Ionization Time of Flight (MALDI-TOF) Mass Spectrometry and Nuclear Magnetic Resonance (NMR) Analysis

Samples were prepared in 2 mg/mL water solution and filtered with a 0.22 μm syringe filter (Pall, Ann Albor, MI, USA). Then, 1 μL of sample was mixed with the same volume of 2,5-dihydroxybenzoic acid (Sigma-Aldrich, St. Louis, MO, USA) as a sample matrix and air-dried for MALDI-TOF analysis. The analysis was performed on an Autoflex III Smart Beam MALDI-TOF mass spectrometer (Bruker, Bremen, Germany) in the positive ion mode. For ^1^H NMR spectral analysis, samples were dissolved in D_2_O (30 mg/mL), and the spectra were carried out on a Bruker AV 500 MHz (Bruker, Karlsruhe, Germany).

### 2.4. Biofilm Formation

*P. aeruginosa* biofilms were cultured in 96-well polystyrene microtiter plates as previously described [16]. Briefly, *P. aeruginosa* was inoculated into LB medium at 28 °C overnight. Then, 100 mL of diluted cell culture (~2 × 10^7^ colony-forming units, CFU) was inoculated into each well of a sterile flat-bottomed 96-well polystyrene micro-titer plate. The microtiter plate was incubated statically at 28 °C for 24 h to allow cell attachment and biofilm formation. *S. aureus* and *L. monocytogenes* biofilms were cultured at 37 °C with TSB medium and LB medium, respectively.

### 2.5. Biofilm Mass and Viability Analysis

In order to evaluate the anti-biofilm activity of COS-Strep conjugates, different treatments were conducted as indicated below. Blank LB broth, LB broth with COS, and LB broth with Strep were used as controls. Moreover, LB broth with the mixture of COS and Strep was also included as a control to determine the necessity of the conjugation. After biofilm formation, the 96-well plate was washed three times with phosphate buffer saline (PBS, pH 7.2) to remove the unattached cells. COS-conjugate and different controls were added into the washed biofilms separately, and the 96-well plates were then incubated at 28 °C for 24 h. Biofilms were washed three times with PBS prior to the analysis. The biofilm mass was determined by the crystal violet assay [17]. The sample was measured for absorbance at 590 nm with a TECAN Infinite M200 PRO multifunction microplate reader (TECAN, Grodig, Austria). To determine the cell viability of the biofilm, MTT [3-(4,5-dimethylthiazol-2-yl)-2,5-diphenyltetrazolium] assay was performed as previously described [18]. Briefly, 100 µL of 500 μg/mL MTT was added into each well and the plate was incubated for 3 h. MTT was then removed and the formed formazan was dissolved in 100 μL dimethyl sulfoxide (DMSO). Optical density (OD) of samples were measured at 490 nm using a TECAN Infinite M200 PRO multifunction microplate reader (TECAN, Grodig, Austria). The IC_50_ of anti-biofilm agents was analyzed using the standard broth microdilution method in accordance with the Clinical and Laboratory Standards Institute (CLSI) guidelines. All tests were performed in six replicates for each treatment. Each assay was performed with three biological repeats. 

### 2.6. Fluorescence Microscopy Assay

One milliliter of *P. aeruginosa* (~2 × 10^7^ CFU) in LB broth was transferred onto 10 × 10 mm glass coverslips (Citoglas, Guangzhou, China) placed on the bottom of the well of 24-well plates and cultivated at 28 °C for 24 h to allow biofilm formation. Unattached cells were removed and coverslips were washed three times with PBS. Formed biofilms were treated with COS-Strep conjugate and controls as indicated above for 24 h at 28 °C. After washing with PBS, 1 µg/mL 4,6-diamidino-2-phenylindole (DAPI, Abbkine, California, America) and 5 µg/mL WGA-FITC (Sigma-Aldrich, St. Louis, MO, USA) were added and incubated in dark for 30 min at 37 °C. Coverslips were then washed and fixed using a 4% paraformaldehyde solution for 30 min at 37 °C. The corresponding fluorescent images were taken by a fluorescent microscope LEICA CTR4000 (Leica, Barnack, Germany).

### 2.7. Cellular Toxicity Assay

Human umbilical vein endothelial cells (HUVECs) were obtained from the American Type Culture Collection (Manassas, VA, USA). The cells were grown in Dulbecco’s modified eagle medium (DMEM) containing 10% fetal bovine serum (FBS) and 100 units/mL penicillin under a 5% CO_2_ atmosphere at 37 °C. The toxicity assay was conducted as following: HUVEC cells were incubated in 96-well plates (3 × 10^3^ cells/well) with Strep or COS-Strep at various concentrations ranging from 50 to 2000 µg/mL for 24 h. The cell toxicity was then evaluated by the MTT assay performed as above. Cell viability (%) was calculated as (absorbance of sample/absorbance of control) × 100.

### 2.8. qRT-PCR Analysis

#### 2.8.1. Isolation of Total RNA

Two milliliters of bacterial cells (~2 × 10^7^ CFU) in LB medium were cultured statically in a 35 × 10 mm style cell culture dish (Corning, New York, NY, USA) for biofilm formation. Drug treatment was conducted as described above. Total RNA was isolated as previously described with some modifications [18,19]. Biofilms were dislodged by 1 mL TRIzon reagent of Ultrapure RNA Kit (CWBio, Beijing, China) and the suspension was collected. The suspension was gently sonicated with a 0.5 cm probe with 10 KHz amplitude for 5 min in a noise isolating chamber JY92-IIN (Scientz, Ningbo, China) to release bacteria from biofilms without mechanical cell disruption. Total RNA was then extracted via acidic phenol–chloroform extraction. The yield and quality of RNA was determined with NanoDrop 2000C (Thermo, New York, NY, USA).

#### 2.8.2. Synthesis of cDNA and RT-PCR

Purified RNA was reverse transcribed into cDNA with HiFiScript cDNA Synthesis Kit (CWBio, Beijing, China). Real-time PCR was performed using the Step One^TM^ Real-Time PCR Instrument Thermal Cycling Block (Applied Biosystems Life Technologies, Foster City, California, USA) with the UltraSYBR Mixture Kit (CWBio, Beijing, China). The expression level of each target gene was normalized to that of the 16S rRNA. Each assay was performed in duplicates with three independent biological repeats. Fold change of mRNA level was calculated according to the 2^−ΔΔCt^ method. Primers used for real-time PCR were listed in Table 1.

#### 2.8.3. Statistical Analysis

Data are presented as means ± SD. A two-tailed Student’s *t*-test was performed for the comparison between two groups and one-way analysis of variance (ANOVA) for multiple group analysis. The *p*-value < 0.05 or 0.01 was considered as statistically significantly different. All data were analyzed using Statistical Product and Service Solutions (SPSS) 13.0 software (SPSS Inc., Chicago, IL, USA).

## 3. Result

### 3.1. Synthesis and Characterization of COS-Strep Conjugates

**Figure 1:**
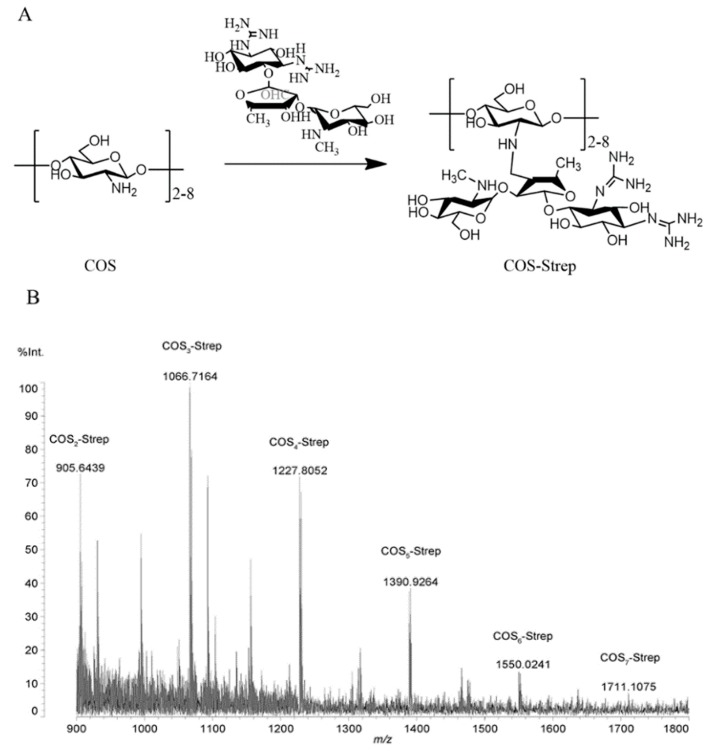
Reaction scheme of the chitosan oligosaccharides-streptomycin (COS-Strep) conjugate synthesis and characterization of synthesized products. Schematic diagram represented the reaction to synthesize of COS-Strep conjugates (**A**). The mass spectrum of COS-Strep conjugates by matrix-assisted laser desorption/ionization time of flight (MALDI-TOF) mass spectrometry (**B**).

The covalent conjugation between streptomycin and chitosan oligosaccharides was achieved by reduction of the resulting Schiff base formed by amino groups in chitosan oligosaccharide and aldehyde groups in streptomycin (Figure 1A), as described [20]. The product of conjugation was determined by MALDI-TOF-MS analysis (Figure 1B). Peaks had an m/z ratio of 905.6439, 1066.7164, 1227.8052, 1390.9264, 1550.0241, 1711.1075, that represented disaccharides (COS2), trisaccharides (COS3), tetrasccharides (COS4), pentasaccharides (COS5), hexaoses (COS6), heptaoses (COS7) coupled with one streptomycin, respectively. The coupling between streptomycin and COS was evidenced by ^1^H NMR analysis (Figure S2). The appearance of signals at 2.57 ppm in the spectrum of COS-Strep was likely attributed to methyl protons (Figure S2A). Weak signals at 9.66 ppm assigned to aldehyde protons in the spectrum of Strep disappeared in that of COS-Strep (Figure S2B). These results strongly suggested the formation of the COS-Strep conjugate.

### 3.2. Inhibitory Effects of COS-Strep against *P. aeruginosa* biofilms

**Figure 2:**
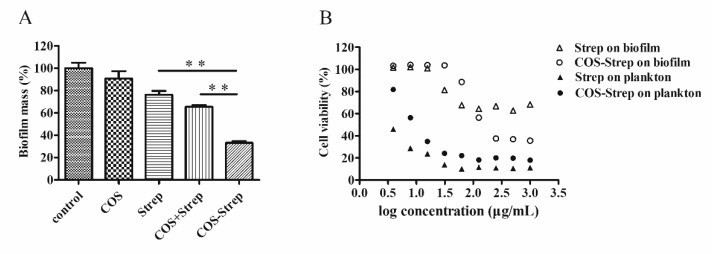
The anti-biofilm activity of COS-Strep conjugates on mature *Pseudomonas aeruginosa* biofilms. *P. aeruginosa* mature biofilms were treated with LB broth containing 250 μg/mL COS, streptomycin, COS-Strep and their mixture (COS + Strep, 250 + 250 μg/mL) for 24 h respectively. Blank medium was used as a control. The biofilm biomass was determined by crystal violet staining and normalized to the control (**A**). Established biofilms were exposed to COS-Strep with a series of concentrations for 24 h and the cell viability of *P. aeruginosa* was determined by MTT assay (**B**). Data are represented as means ± SD (*n* = 6). * *p* < 0.05 or ** *p* < 0.01.

Established *P. aeruginosa* biofilms were treated with COS-Strep to evaluate the anti-biofilm activity of the conjugate. COS-Strep had strongest capability in removing mature biofilms with a minimum effective concentration of 250 µg/mL; COS and Strep had no or weak anti-biofilm activity at 250 µg/mL (Figure 2A). Moreover, COS-Strep showed high efficacy on killing *P. aeruginosa* cells in the cell viability test. The IC_50_ value of COS-Strep is 88.35 µg/mL, which is 11-fold lower than that of streptomycin (>1000 µg/mL). For planktonic bacteria, the IC_50_ value of COS-Strep was slightly higher than that of streptomycin (Figure 2B). To be noted, a simple mixture with COS did not improve the anti-biofilm activity of streptomycin (Figure 2A). These results suggested that coupling with COS enhanced the antimicrobial efficiency of streptomycin against biofilm of *P. aeruginosa*.

### 3.3. The Degree of Polymerization of COS Influenced the Anti-Biofilm Efficacy of COS-Strep Conjugates

**Figure 3:**
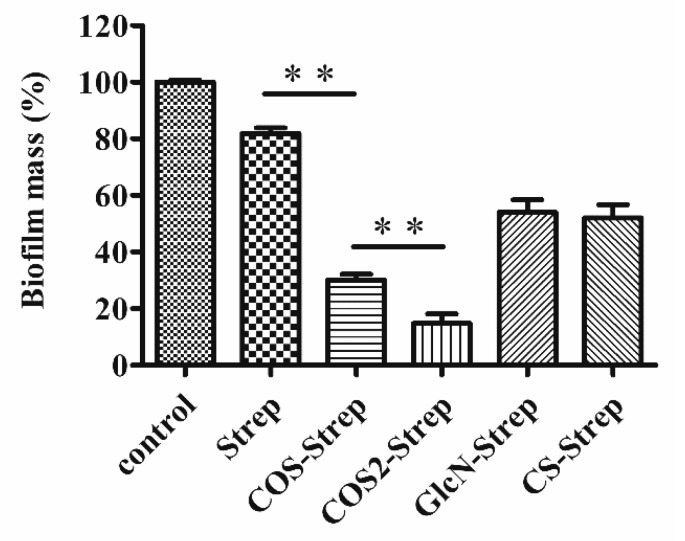
The anti-biofilm activity of COS-Strep with different polymerization degrees. Mature *P. aeruginosa* biofilms were exposed to 250 μg/mL COS-Strep, COS2-Strep, GlcN-Strep and chitosan-streptomycin conjugate (CS-Strep) for 24 h. The residual biofilm mass relative to the control was assessed by the crystal violet staining assay. Data are represented as means ± SD (*n* = 6). * *p* < 0.05; ** *p* < 0.01.

Studies showed that biological activities of COS were largely dependent on its degree of polymerization (DP) [2,21,22]. To investigate whether the DP of COS affects the anti-biofilm capacity of COS-Strep conjugates, two COS products with different DP, including COS (DP 2~8) and COS2 (DP 2~20) as well as Glucosamine and chitosan were conjugated with streptomycin to produce COS-Strep, COS2-Strep, GlcN-Strep and CS-Strep conjugates respectively. Four synthesized conjugates showed enhanced anti-biofilm activities against biofilms than streptomycin alone at 250 μg/mL (Figure 3). Moreover, two COS-streptomycin conjugates, COS-strep and COS2-Strep, removed more than 70% biofilms mass, while streptomycin was only capable of removing 22%.

On the contrary, GlcN-Strep and CS-Strep conjugates only slightly enhanced the anti-biofilm activity compared to streptomycin (Figure 3). Among the two COS-Strep conjugates, COS2-Strep exhibited the highest anti-biofilm activity. These results indicated that the anti-biofilm activity of streptomycin can be enhanced by conjugating with COS rather than its structurally similar polymers or monosaccharide component. Furthermore, the anti-biofilm activity of COS-Strep conjugates was affected by the degree of polymerization of COS used in the conjugation process. 

### 3.4. Coupling with COS Did not Improve the Anti-Biofilm Activity of Streptomycin on *S. aureus* and *L. monocytogenes*

**Figure 4:**
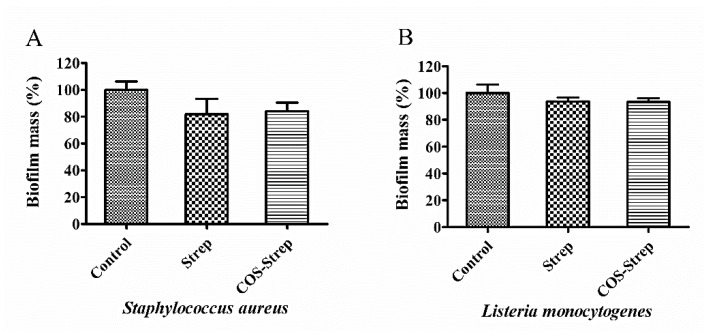
The anti-biofilm activity of COS-Strep conjugate on Gram-positive bacterial biofilms. *Staphylococcus aureus* (**A**) and *Listeria monocytogenes* (**B**) mature biofilms were exposed to COS-Strep for 24 h at 250 μg/mL. The biofilm mass was determined by crystal violet staining assay. Data are represented as means ± SD (*n* = 6).

To investigate the anti-biofilm activity of COS-Strep on other bacteria, we assessed the activity of COS-Strep on mature biofilms of *S. aureus* and *L. monocytogenes*, which are Gram-positive opportunistic human pathogens. COS-Strep or Strep alone did not show inhibition effects against biofilms of tested strains at 250 μg/mL (Figure 4).

### 3.5. Cellular Toxicity

**Figure 5:**
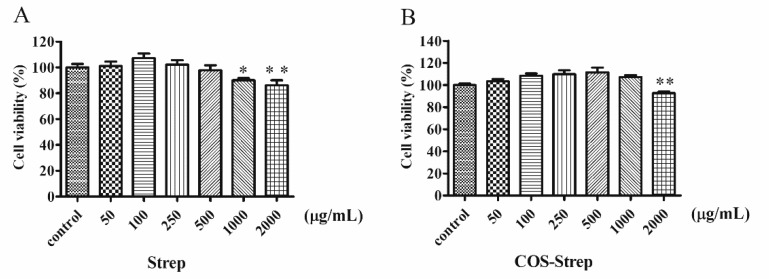
The cell toxicity of streptomycin (**A**) and COS-Strep (**B**) on HUVEC cells. Data are represented as means ± SD (*n* = 8). * *p* < 0.05 or ** *p* < 0.01, compared to the control group.

HUVEC cells were used to determine potential side effects of the COS-Strep conjugate in cell viability analysis. Same as streptomycin (Figure 5A), COS-Strep had no obvious effects on the growth of the mammalian cell strain at 1 mg/mL (Figure 5B).

### 3.6. Influence on *P. aeruginosa* Biofilm Related Gene Expressions under the COS-Strep Conjugate Treatment

**Figure 6:**
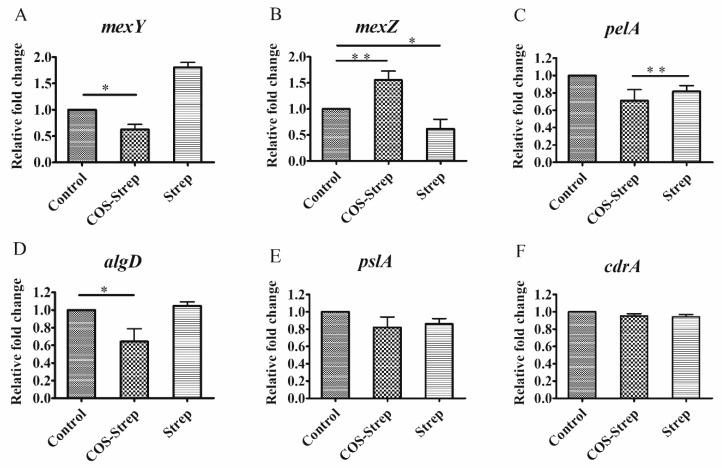
Differences in the expression levels of biofilm-related genes of *P. aeruginosa*. *mexY* (**A**), *mexZ* (**B**), *pelA* (**C**), *algD* (**D**), *pslA* (**E**), *and cdrA* (**F**) in *P. aeruginosa* following COS-Strep or streptomycin treatment. Data are represented as means ± SD (*n* = 3). * *p* < 0.05; ** *p* < 0.01.

To explore the mechanism of the antibacterial activity of COS-Strep on *P. aeruginosa* biofilms, expression levels of several genes related to biofilm formation or drug-resistance were determined. Genes including *pslA*, *pelA*, and *algD*, which are related to biosynthesis of biofilm exopolysaccharides Psl, Pel, and alignate [23,24]; *MexY*, and *mexZ* which encode proteins involved in MexX-MexY drug efflux pump system [25,26]; and *cdrA* which encode proteins to mediate bacterial aggregation and biofilm adherence [27], were selected as targets. Streptomycin treatment greatly upregulated the expression of *mexY* by 1.9 fold in biofilm cells, suggesting the activation of the MexX-MexY drug efflux pump system (Figure 6A). On the contrary, COS-Strep treatment slightly down-regulated the expression of *mexY* (Figure 6A), likely through upregulating the expression of its suppressor, *mexZ* (Figure 6B). Thus, COS-Strep treatment did not upregulate or even suppress the MexX-MexY system which was activated by streptomycin. Genes *pelA* and *algD* were also down-regulated by 0.68 and 0.75 fold, respectively with COS-Strep treatment compared to the control or streptomycin treated group (Figure 6C,D). On the other hand, the expression level of *pslA* and *cdrA* remained unaffected under COS-Strep treatment (Figure 6E,F). Therefore, the anti-biofilm activity of COS-Strep might be because of its ability to suppress the activation of the drug efflux pump system and the biosynthesis of specific exopolysaccharides. 

### 3.7. COS-Strep Treatment Reduced Biofilm Exopolysaccharides of *P. aeruginosa*

**Figure 7:**
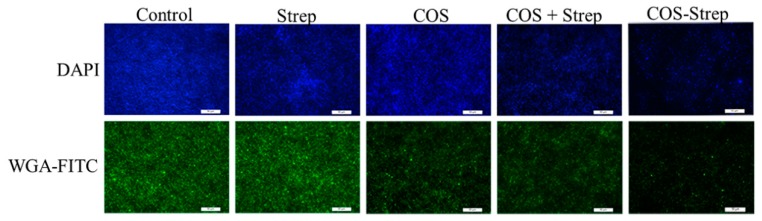
COS-Strep affected biofilm exopolysaccharides of *P. aeruginosa*. Drug treated biofilms were observed by fluorescence image assay. Shown are single-channel images of signal derived from staining with 4,6-diamidino-2-phenylindole (DAPI) (nucleus, blue, upper panel) and WGA-FITC (exopolysaccharide, green, lower panel). Scale bar, 50 μm.

In order to investigate the effect of COS-Strep on biofilm exopolysaccharides, we stained the biofilm with FITC labeled wheat germ agglutinin (WGA) lectin which bound to exopolysaccharide, and DAPI. We observed the reduction of signals with WGA-FITC and DAPI staining after COS-Strep treatment, suggesting its influence on both biofilm cell viability and exopolysaccharide (Figure 7).

## 4. Discussion

Streptomycin is a common aminoglycoside antibiotic which is used to treat a number of bacterial infections. However, pathogens in biofilms could greatly increase their drug resistance. It has been previously reported that chitosan coupling with streptomycin (CS-Strep) could eradicate biofilms of several Gram-positive organisms, but barely affect that of Gram-negative organisms such as *P. aeruginosa* [11]. However, the underlying molecular mechanism of the CS-Strep remained unclear. Moreover, poor water-solubility of CS-Strep might restrict its applications on anti-infection therapy. In this study, we prepared the chitosan oligosaccharide-streptomycin conjugate (COS-Strep), which has an improved water-solubility. Our data showed that the COS-Strep conjugate was much more effective in eradicating established biofilms of *P. aeruginosa* than streptomycin alone or their simple mixture (Figure 2). The efficacy of the anti-biofilm activity was affected by the DP of COS used for conjugation (Figure 3). The activity of COS-Strep on the *P. aeruginosa* biofilm was likely through inhibiting response of the MexX-MexY drug efflux pump system and synthesis of biofilm exopolysaccharides (Figure 6). 

Surprisingly, unlike CS-Strep, the COS-Strep conjugate did not show an obvious anti-biofilm activity on Gram-positive organisms such as *S. aureus* and *L. monocytogenes* (Figure 4). However, COS-Strep effectively inhibited against *P. aeruginosa* biofilms (Figure 2), suggesting their opposite effects on tested organisms. Although COS and chitosan shared structural similarity, both showed anti-biofilm activity when conjugating with streptomycin, the mechanism behind the antimicrobial activity of the two conjugates might be different. Previous studies on the antibacterial activity of chitosan and COS indicated that chitosan had stronger bactericidal effects with gram-positive bacteria than gram-negative bacteria [28], while COS showed better activity against gram-negative bacteria [29]. These diverse activities might be relevant to the difference in the composition of cell walls and biofilm matrix of different organisms or the antibacterial action mode of COS and chitosan. The selective anti-biofilm activity of the COS-Strep conjugate on *P. aeruginosa* was likely determined by the glycan part of the conjugate, especially considering that streptomycin was not a suitable antibiotic for treatment of *P. aeruginosa* infection. 

*P. aeruginosa* is a major clinical opportunistic pathogen. There is an increasing awareness of the important role of *P. aeruginosa* biofilm infections associated with specific tissue and implants such as the mucus plugs of the cystic fibrosis (CF) lungs, catheters, and contact lenses [30,31]. Antibiotic treatments for *P. aeruginosa* sometimes are inefficient due to its ability to form biofilms on various organic and inorganic surfaces. Studies have shown that the MexX-MexY drug efflux pump system plays a vital part in the intrinsic resistance of *P. aeruginosa* to aminoglycosides. The MexX-MexY system was regulated by *mexZ* which was a repressor of *mexY* expression [25,26]. To explore whether the antibacterial activity of COS-Strep on *P. aeruginosa* biofilms was through affecting MexX-MexY drug efflux pump system, the expression levels of several genes related to MexX-MexY drug efflux pump were determined under the treatment of COS-Strep. The results showed that COS-Strep did not induce the activation of the MexX-MexY system compared to streptomycin, (Figure 6). This result suggested that the COS-Strep conjugate suppressed the response of this drug-resistance machinery. The mechanism behind this activity requires further investigation.

We also found that expressions of exopolysaccharide biosynthesis genes were influenced under the COS-Strep treatment. In *P. aeruginosa* biofilms, Psl, Pel and alginate exopolysaccharides served as key structural components of the biofilm matrix [32]. Previous studies indicated that Psl and alginate were required for formation of biofilms, whereas Pel played an important role in controlling biofilm cell density and/or the compactness of biofilms [33]. Our studies showed that the expression of *pelA* and *algD* genes were suppressed under the treatment of COS-Strep (Figure 6C,D), but not *pslA* and *cdrA* (Figure 6E,F). CdrA, a biofilm matrix protein, is related to extracellular adhesion and promotes tight cellular interactions in biofilm aggregates [27]. The result indicated COS-Strep might impair the structural integrity of the biofilm by inhibiting the biosynthesis of Pel and alginate exopolysaccharides. Pel is a positively charged polysaccharide composed of partially acetylated 1-4 glycosidic linkages of *N*-acetylgalactosamine and *N*-acetylglucosamine [34]. WGA lectin usually recognizes *N*-acetylglucosamine-containing glycans. To further explore the possible influence of COS-Strep on biofilm exopolysaccharides, we determined the biofilm structure after drug treatment under a fluorescence microscope, using FITC (green fluorescent bioconjugates) labeled WGA lectin. Our results showed a significant reduction of green fluorescent signals after treatment with COS-Strep (Figure 7). It further supported that COS-Strep affected the structural integrity of biofilm exopolysaccharides. 

## 5. Conclusions

In summary, our result highlighted that chitosan oligosaccharide coupling greatly increased the susceptibility of *P. aeruginosa* biofilms to streptomycin. COS-Strep treatments might suppress the response of the drug efflux pump system and inhibited the biosynthesis of Pel and alginate exopolysaccharides. Given chitosan oligosaccharide gain considerable attention as a biomaterial, due to its good water-solubility and low toxicity, this novel strategy might open up a new avenue to overcome the inherent resistance of biofilms to antibiotics, and come into wide use for combating biofilm-related problems in industrial and medical areas.

## Figures and Tables

**Table 1:** Primers used for the qRT-PCR.

Primer	Sequence (5′-3′)
algD-F	AGAAGTCCGAACGCCACA
algD-R	TCCAGCTCGCGGTAGAT
pelA-F	CCTTCAGCCATCCGTTCTTCT
pelA-R	TCGCGTACGAAGTCGACCTT
pslA-F	AAGATCAAGAAACGCGTGGAAT
pslA-R	TGTAGAGGTCGAACCACACCG
mexY-F	TTACCTCCTCCAGCGGC
mexY-R	GTGAGGCGGGCGTTGTG
mexZ-F	TTACCTCCTCCAGCGGC
mexZ-R	GTGAGGCGGGCGTTGTG
16S rRNA-F	AACCTGGGAACTGCATCCAA
16S rRNA-R	CTTCGCCACTGGTGTTCCTT

## Supplementary Materials

The following are available online at http://www.mdpi.com/1660-3397/17/1/43/s1. Figure S1. The results of HPLC analysis of COS (DP2-8) and COS (DP 2-20) (A) and LC-MS results of COS and COS2; Figure S2. ^1^H NMR spectra of COS, streptomycin, COS+Strep and COS-Strep conjugates. The Freeze-dried COS, Strep, the COS-Strep conjugate and a mixture of two molecules (mass ratio 1:1) were dissolved in deuterated water to a final concentration of 30mg/mL respectively. The spectra were recorded at 298 K in deuterium oxide on a Varian VNMRS-500 NMR spectrometer. Red arrow represented the methyl protons singal at 2.57 ppm in COS-Strep conjugates (A). Red arrow represented aldehyde proton singal at 9.66 ppm that was the functional group of streptomycin (B).
