# Supplementary material for: Synthesis and Evaluation of a Chitosan Oligosaccharide-Streptomycin Conjugate against Pseudomonas aeruginosa Biofilms
Source: Mar Drugs. 2019 Jan 10;17(1):43. doi: 10.3390/md17010043 (PMC6356912; doi:10.3390/md17010043)
Supplement: Supplementary file 1 [file marinedrugs-17-00043-s001.pdf]

Synthesis and Evaluation of a Chitosan Oligosaccharide-Streptomycin Conjugate against *Pseudomonas aeruginosa* Biofilms

Ruilian Li [1,2], Xianghua Yuan [3], Jinhua Wei [2], Xiafei Zhang [3], Gong Cheng[2], Zhuo A. Wang [2,*] and Yuguang Du [2,*]

[1] University of Chinese Academy of Sciences, Beijing 100049, China; rlli@ipe.ac.cn
[2] Key Laboratory of Biopharmaceutical Production & Formulation Engineering, PLA and State Key Laboratory of Biochemical Engineering, Institute of Process Engineering, Chinese Academy of Sciences, Beijing 100190, China; jhwei@ipe.ac.cn (J.W.); gcheng@ipe.ac.cn
[3] (G.C.) College of Life Science, Sichuan Normal University, Chengdu 610101, China; lemonlyty@sohu.com (X.Y.); feifei_2016@126.com (X.Z.)
* Correspondence: wangzhuo@ipe.ac.cn (Z.A.W.); ygdu@ipe.ac.cn (Y.D.); Tel./Fax: +86-10-8254-5070 (Z.A.W. & Y.D.)

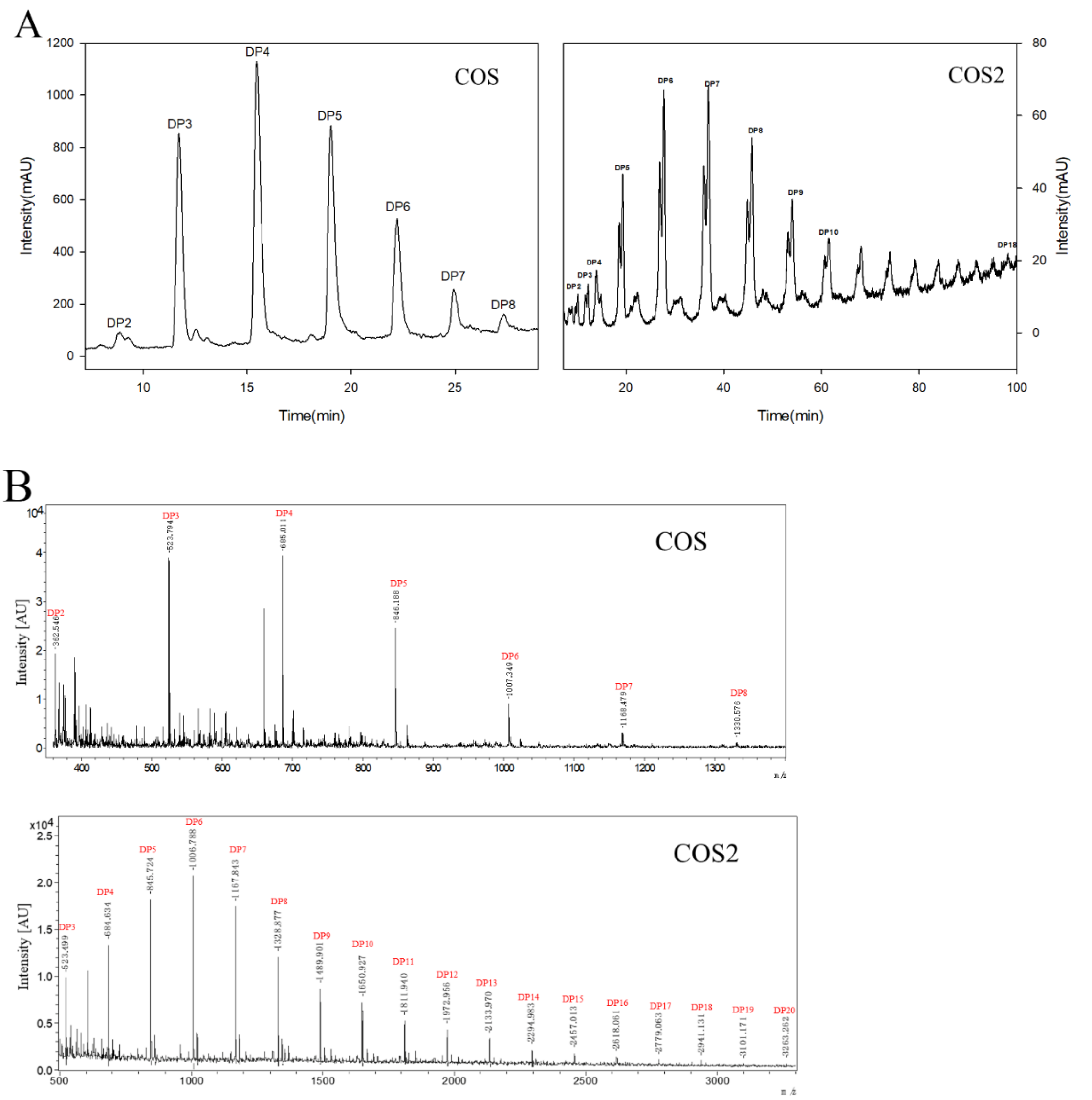

Figure S1. The results of HPLC analysis of COS (DP2-8) and COS (DP 2-20) (A) and LC-MS results of COSand COS2.

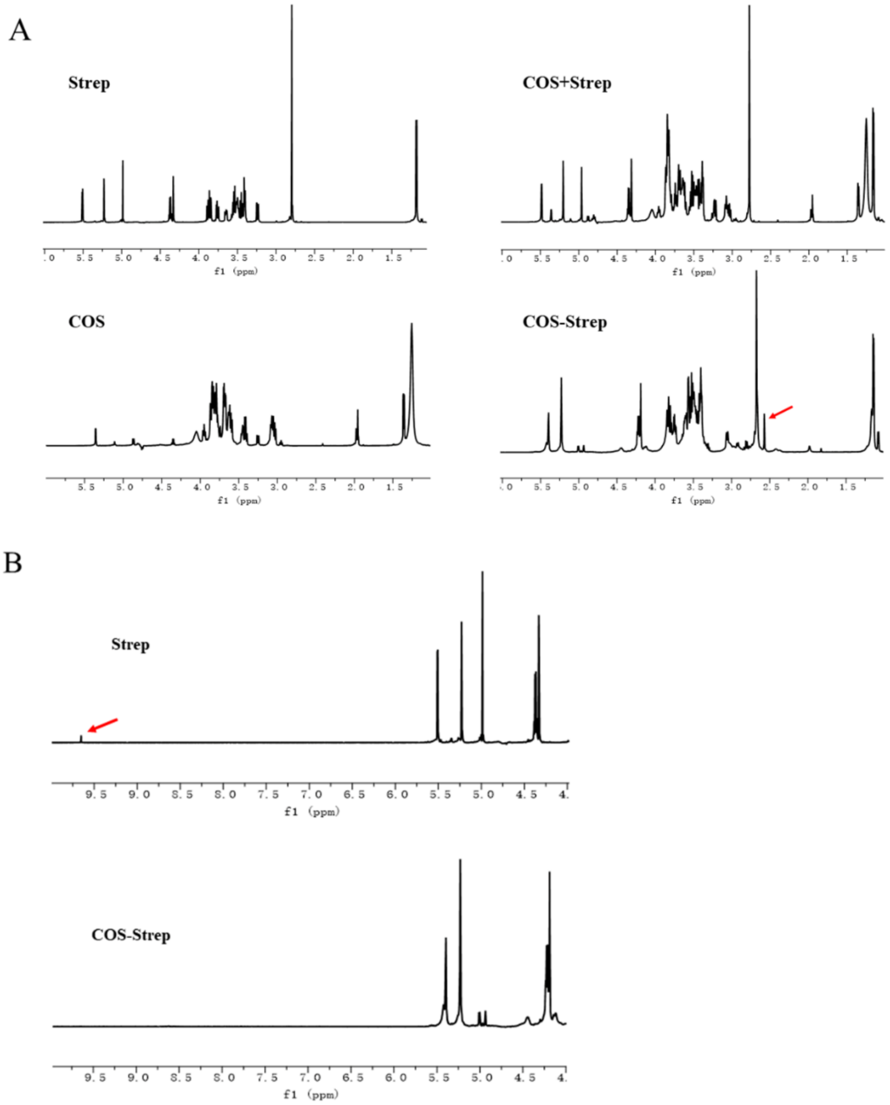

Figure S2. ^{1}H NMR spectra of COS, streptomycin, COS+Strep and COS-Strep conjugates. The Freeze-dried COS, Strep, the COS-Strep conjugate and a mixture of two molecules (mass ratio 1:1) were dissolved in deuterated water to a final concentration of 30mg/mL respectively. The spectra were recorded at 298 K in deuterium oxide on a Varian VNMRS-500 NMR spectrometer. Red arrow represented the methyl protons singal at 2.57 ppm in COS-Strep conjugates (A). Red arrow represented aldehyde proton singal at 9.66 ppm that was the functional group of streptomycin (B).
